# Assessment of facility readiness for implementing the WHO/UNICEF *standards for improving quality of maternal and newborn care in health facilities* – experiences from UNICEF’s implementation in three countries of South Asia and sub-Saharan Africa

**DOI:** 10.1186/s12913-018-3334-0

**Published:** 2018-07-09

**Authors:** Alexander Manu, Shams Arifeen, John Williams, Edward Mwasanya, Nabila Zaka, Beth Anne Plowman, Debra Jackson, Priscilla Wobil, Kim Dickson

**Affiliations:** 1 London School of Hygiene & Tropical Medicine, London, UK; 2 Centre for Maternal and Newborn Health, Liverpool School of Tropical Medicine, Pembroke Place, Liverpool, L3 5QA UK; 3 International Centre for Diarrhoeal Diseases Research, Dhaka, Bangladesh; 4 Navrongo Health Research Centre, Navrongo, Ghana; 5 National Institute for Medical Research, Dar es Salaam, Tanzania; 6 United Nations Children’s Fund/HQ, New York, USA; 7 United Nations Fund for Population, Freetown, Sierra Leone

## Abstract

**Background:**

There is a global drive to promote facility deliveries but unless coupled with concurrent improvement in care quality, it might not translate into mortality reduction for mothers and babies. The World Health Organization published the new “*Standards for improving quality of care for mothers and newborns in health facilities”* but these have not been tested in low- and middle-income settings. UNICEF and its partners are taking the advantage provided by the Mother and Baby Friendly Hospital Initiative in Bangladesh, Ghana and Tanzania to test these standards to inform country adaptation. This manuscript presents a framework used for assessment of facility quality of care to inform the effect of quality improvement interventions.

**Methods:**

This assessment employed a quasi-experimental design with pre-post assessments in “implementation” and “comparison” facilities-the latter will have no quality improvement interventions implemented. UNICEF and assessment partners developed an assessment framework, developed uniform data collection tools and manuals for harmonised training and implementation across countries. The framework involves six modules assessing: facility structures, equipment, drugs and supplies; policies and guidelines supporting care-giving, staff recruitment and training; care-providers competencies; previous medical records; provider-client interactions (direct observation); and client perspectives on care quality; using semi-structured questionnaires and data collectors with requisite training. In Bangladesh, the assessment was conducted in 3 districts. In one "intervention" district, the district hospital and five upazilla health complexes were assessed. similar number of facilities were assessed each two adjoining comparison districts. In Ghana it was in three hospitals and five health centres and in Tanzania, two hospitals and four health centres. In the latter countries, same number of facilities were selected in the same number of districts to serve for comparison. Outcomes were structured to examine whether facilities currently provide services commensurate with their designation (basic or comprehensive emergency obstetric and newborn care). These outcomes were stratified so that they inform intervention implementation in the short-, medium- and long-term.

**Conclusion:**

This strategy and framework provides a very useful model for supporting country implementation of the new WHO standards. It will serve as a template around which countries can build quality of care assessment strategies and metrics to inform their health systems on the effect of QI interventions on care processes and outcomes.

## Background

The time around birth (from the onset of labour up to the first week after delivery) presents the greatest risk in the life of a mother and her newborn. [1–3] Approximately 280,000 maternal deaths, about 4 million stillbirths and early neonatal deaths still occur annually mainly in low and middle-income settings (LMICs). [4–7] There is a global drive to promote facility deliveries [8, 9] but unless coupled with health system strengthening to improve quality of care within facilities, the increased access will not likely translate into reduction in maternal, fetal and newborn deaths. [10] Universal health coverage (UHC) and quality of care (QoC) are now recognised as the two main pillars for addressing these preventable deaths. [11]

UNICEF and the World Health Organization (WHO) are leading global efforts to consolidate and further enhance the gains made in child survival through many initiatives. The flagship action plan, Every Newborn Action Plan (ENAP), was ratified by the 67th World Health Assembly in 2014. ENAP is the strategy to implement an Every Mother Every Newborn (EMEN) Quality Improvement (QI) initiative in support of United Nations Secretary-General’s Every Woman Every Child movement. Subsequent developments on quality of care agenda has led to the recent launch of Quality Equity Dignity network with nine first phase countries. Bangladesh, Ghana and Tanzania are included in the list of fore-runner countries. A key component of ENAP is to improve health facility quality of care for mothers and newborns (http://www.who.int/pmnch/about/governance/partnersforum/enap_committments/en/ [http://www.who.int/pmnch/about/governance/partnersforum/enap_committments/en/). WHO/UNICEF identified three target intervention areas for quality improvement (QI): Clinical - that assess quality of clinical care content; Patients’ rights - ensuring dignified and respectful care for mothers and newborns; and Cross-cutting issues – securing an enabling physical environment and governance structures for quality care. [11] These were translated into ten(10) interlinked core quality standards (with indicators) aimed at reducing mortality and severe morbidity; improving access to services; and ensuring safety of mothers and newborns. These standards define sets of “criteria” outlining the elements that need to be in place to meet them. They have, subsequently, been refined to form the eight WHO standards for improving quality of maternal and newborn care in health facilities [12] (Table 1). However, they have not been tested within health facilities to assess feasibility, acceptability and potential impact on pregnancy and birth outcomes in LMICs. This testing is a critical step to facilitate global roll-out and adaptation of the standards.

**Table 1:** Relationship between the EMEN standards and the eight WHO quality standards

The 3 domains and how they translated into the initial 10 quality of care standards	Current WHO Standards for improving quality of maternal & newborn care in health facilities
Clinical Care 1. Evidence-based safe antenatal care is provided. 2. Evidence-based safe care is provided during labor and childbirth. 3. Evidence-based safe postnatal care is provided for all mothers and the newborns. Patients’ Rights 4. Human rights are observed and the experience of care is dignified and respectful for every woman and newborn. Crosscutting 5. A governance system is in place to support the provision of quality maternal and newborn care. 6. The physical environment of the health facility is safe for providing maternal and newborn care. 7. Qualified and competent staff are available in adequate numbers to provide safe, consistent and quality maternal and newborn care. 8. Essential drugs, supplies and functional equipment and diagnostic services are consistently available for maternal and newborn care. 9. Health information systems are in place to manage patient clinical records and service data. 10. Services are available to ensure continuity of care for all pregnant women, mothers and newborns.	Standard 1: Every woman and newborn receive routine, evidence-based care and management of complications during labour, childbirth and the early postnatal period, according to WHO guidelines. Standard 2: The health information system enables use of data to ensure early, appropriate action to improve the care of every woman and newborn. Standard 3: Every woman and newborn with condition(s) that cannot be dealt with effectively with the available resources is appropriately referred. Standard 4: Communication with women and their families is effective and responds to their needs and preferences. Standard 5: Women and newborns receive care with respect and preservation of their dignity. Standard 6: Every woman and her family are provided with emotional support that is sensitive to their needs and strengthens the woman’s capability. Standard 7: For every woman and newborn, competent, motivated staff are consistently available to provide routine care and manage complications. Standard 8: The health facility has an appropriate physical environment, with adequate water, sanitation and energy supplies, medicines, supplies and equipment for routine maternal and newborn care and management of complications.

UNICEF has partnered with the Bill and Melinda Gates Foundation (BMGF) to support ministries of health in Bangladesh, Ghana and Tanzania to implement a 3-year maternal, newborn care and breastfeeding partnership called the Mother and Baby Friendly Health Facility Initiative (MBFHI) in identified regions within the countries. The MBFHI provides a platform to test the EMEN-QI standards and inform adaptations required for specific contexts. Testing of such high impact standards therefore necessitates rigorous evaluation. This manuscript describes the design, conceptual framework, and harmonised implementation of the assessment model for the EMEN-QI initiative including the development of harmonised and uniform procedures and data collection tools across countries and sites. It also details the baseline assessment conducted before commencement of QI interventions and makes a case for the evaluation’s utility in similar settings in South Asia and sub-Saharan Africa.

## Methods

### Overview of the assessment for testing the implementation of WHO standards for improving quality of maternal and newborn care (EMEN-QI initiative)

#### Where did we begin? Development of the assessment model

In mid-2015, UNICEF/WHO coordinated the development of ten EMEN maternal and newborn care quality improvement standards. UNICEF, as the lead partner for the implementation, proposed using the MBFHI platform to test the implementation of these standards. UNICEF worked with Ministries of Health in the three countries to identify sites and facilities where the EMEN-QI model will be implemented (“intervention” facilities) as well as independent institutions to carry out the evaluation/assessment. The International Centre for Diarrhoeal Diseases Research, Bangladesh (ICDDR,B), Navrongo Health Research Centre (NHRC) of the Ghana Health Service (GHS), and the National Institute of Medical Research, Tanzania (NIMR) were selected to conduct the independent assessment of the implementation. To ensure uniformity in key aspects of the assessments across countries, UNICEF convened the team in New York in February 2016 to agree on harmonized process and impact outcomes, indicators, determine the overall objectives, design, strategy, and timelines for the assessment.

#### WHO standards for improving health facility quality of Care for Mothers and Newborns [12]

These standards were developed in accordance with the standard operating procedures contained in the WHO handbook for guideline development. [13] A guideline steering group was constituted to lead the development which involved the following steps:

1. Scoping meeting: The steering group constituted experts to define the research questions and identify the gaps which the standards are expected to address. The experts identified definition of quality in the context of maternal and newborn care, models for quality improvement and conceptual frameworks supporting these and effective strategies for clinical and health care improvements.
2. Evidence retrieval and synthesis: Searches were conducted to summarize evidence to answer the questions identified in the scoping exercise. The evidence was graded for quality and summarized.
3. Technical guideline development group and consensus-building: A team of experts were convened for technical consultation to discuss the evidence retrieved and synthesized to formulate the framework and the standards statements for quality improvement in health facilities.
4. Development of measures: A Delphi process was used to collect, collate, and build consensus around the quality measures addressing each of the standards.

These processes culminated in the initial ten and fine-tuned to the published eight WHO standards for improving quality of care for mothers and newborns in health facilities as shown in Table 1. [12]

#### The overall design of the assessment

**Fig. 1:**
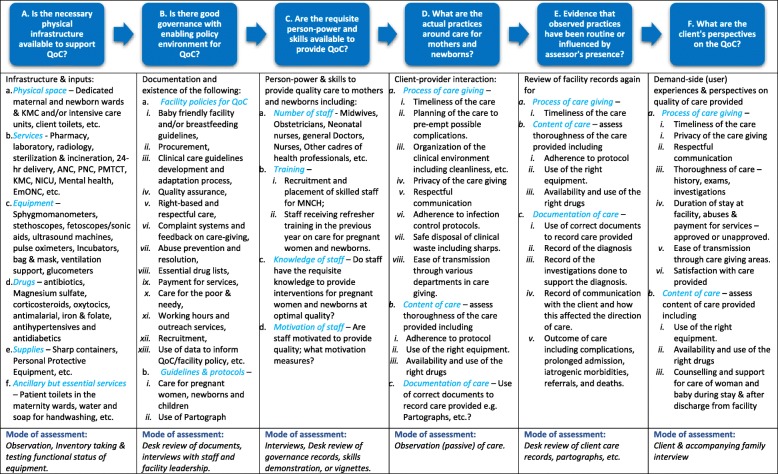
EMEN Quality of care (QoC) assessment framework: A composite to assess inputs, processes, and outputs together with user-perspective

The assessment of the facility quality of care improvement adopts a quasi-experimental pre-post or “before and after” design in “intervention” and “comparison” groups using mixed methods (qualitative and quantitative). In the intervention facilities, the EMEN-QI models are being implemented to improve quality of care for mothers and newborns. “Comparison” facilities, where the EMEN-QI initiative are not being implemented, were selected using standard criteria including the facility’s level in services hierarchy (hospital, health centre/Upazil or other), designation based on expected services (Comprehensive or Basic Emergency Obstetric Care (CEmOC or BEmOC)), annual caseload, proximity and contextual issues. Baseline and Endline cross-sectional assessment of quality of care within the facilities was conducted using a harmonised framework (Fig. 1). The assessment therefore compares differences in inputs, processes, and outcomes before and after the implementation of the EMEN-QI initiative in the “intervention” facilities. Data from the “matched” comparison facilities will be used to control/adjust for changes in quality of care due to other developments within the health systems in the respective countries, independent of the EMEN-QI initiative.

In the design, the assessment teams will also conduct quarterly monitoring visits to “intervention” facilities to assess progress of implementation of EMEN-QI standards. In these monitoring visits, they will elicit challenges encountered by the implementation teams within the facilities, document how they are being resolved and any useful lessons learned to inform future programme roll-out. This process monitoring data will also help explain possible mechanisms by which the interventions result in the outcomes at Endline–whether positive or negative.

#### Aim & Objectives

The overall aim of the assessment is to determine the impact of the EMEN facility QI interventions on facility care delivery processes and outcomes for mothers and babies. The objectives are detailed in Table 2 but in summary, the assessment:

i. Assesses existing facility resources (physical, human) for quality care provision;
ii. Determines the extent to which quality care has been institutionalized within facilities;
iii. Evaluates how quality improvement inputs and process are translated into patient care and outcomes for clients;
iv. Documents lessons learned to inform future implementation of the initiative.

#### Study settings and facilities

In Bangladesh, the EMEN-QI model is being implemented in Kurigram (Rangpur division), a district with nine sub-districts (Upazilas) in the northern border of Bangladesh. It has a projected population of 2,069,273 [14] and was purposively selected because it has the least number of health-related interventions. The EMEN-QI initiative is being implemented in the Kurigram district hospital along with four Upazila health complexes as shown in Table 3. Two adjoining districts (Lalmonirhat and Gaibandha), each with a district hospital, and four Upazila health complexes will serve as comparison facilities for the assessment.

**Table 2:** Objectives of the overall evaluation of the EMEN facility QI

i. To assess the “structural readiness”, including physical infrastructure, human resources, care provider skills, equipment, drugs and supplies (i.e. ‘what was there to use to provide quality care for the patient’), of targeted health facilities in South Asia and sub-Saharan Africa for implementing EMEN QI initiative	
ii. To determine to what extent components of the EMEN QI standards have been “institutionalized” within these facilities and assess the integrity (comparing with written protocols) and progress of implementation by estimating the proportion of the targeted facilities that met at least 75% of the EMEN standards and criteria at Endline.	
iii. To examine improvements in perinatal clinical outcomes for mothers and newborns including client perceptions and satisfaction with service quality (“what was the result for clients”) and record-keeping practices around these.	
iv. To document and describe the key lessons learned to inform future guidance for the implementation of the EMEN QI model.	

In Ghana, the initiative is being implemented in the Upper East Region (UER) of northern Ghana. The region has 13 administrative districts, a projected population of 1,109,338, representing 4.0% of the population of Ghana. [15] The region has shown a lot of promise in improving perinatal outcomes as demonstrated in the perinatal outcomes in the 2014 Ghana Demographic and Health Survey, though the results were obtained without adjustment for sampling design (unweighted results). [16] Four target districts (Bawku & Bolgatanga municipalities, Bongo, and Kassena-Nankana West, KNW) were selected by the Ghana Health Service for the implementation of the MBFHI intervention. Table 3 shows the public hospitals and health centres purposively selected as “intervention” facilities by the UER health administration based on structural readiness, high volume of deliveries and good leadership. Comparison districts were also selected using criteria described above.

In Tanzania, the Njombe region with a projected population of 724,836 (projected from the 2012 population census), [17] with high levels of maternal and newborn mortality in health facilities, was selected for the EMEN-QI initiative. Table 3 shows there are 14 healthcare facilities in Ludewa and Wanging’ombe districts, including hospitals, health centres, and dispensaries were selected as “intervention” facilities, implementing these standards. Comparison facilities from the Njombe TC and Makete districts were also selected for the assessment.

### The baseline assessment

The baseline assessment was designed to be a comprehensive assessment of the state of quality of care existing within health facilities prior to the adoption of the EMEN-QI standards. The wide scope of the baseline assessment is to:

1. Develop and implement a model baseline assessment strategy and tools that can be adapted and used in health facilities implementing the EMEN-QI model in LMICs.
2. Implement the model baseline assessment of the EMEN facility QI model within health facilities in the three countries to
  - Assess existing infrastructure and resources (human, policy environment, existing quality improvement initiatives and other) to provide quality of care.
  - Examine service provision in the facilities and its quality from user perspectives
  - Identify quality gaps to inform the focus, measure progress and document key lessons for future adaptation of the strategy in similar settings.

#### Expected outcomes for the baseline assessment

The outcomes will include an inventory of existing inputs, on-going processes and impact outcomes of these processes and are tailored to reflect the main objectives of the overall EMEN-QI assessment as in Table 2.

#### Implementation strategy for the baseline assessment

The fieldwork training manual covering the design and details for the baseline assessment has been published. [18] The EMEN-QI assessment teams determined what will be adequate contact time with each facility so that interviewers will have a wider sampling frame for health worker and client respondents. This was based on the anticipated number and mix of these respondents especially in large facilities where workers run weekly rotas. They agreed on 14 days and nights of data collection (including weekends) per facility so that the assessment can cover a wider range of maternal and newborn health complications for which management protocols and practices could be observed. The team considered the observation of care provision to clients who visited facilities during the night as critical because, based on physiological and other reasons, a good proportion of labour and deliveries occur at night and health provider responses to complications that occur at the night may differ from when they occur during the day. These observed outcomes of care provision will feed into the baseline quality improvement indicators of the assessment. The 14 days also enables the team to follow-up and document the experiences of some clientele who present in labour until their discharge from the facility, even if they had caesarean delivery and it does not needlessly delay the start of implementation of the QI interventions.

#### Data collectors

Interviewers to collect data in the facilities were organised in teams of four (for hospitals) or three (for health centres or upazila health complexes) members comprising at least one clinically-trained professional and one social scientist. It was determined that study clinicians must have relevant clinical experience especially exposure to maternal and newborn health care so that they will understand and accurately interpret client-provider interactions and document responses to clinical vignettes during health provider interviews. The main role of the social scientist was to conduct the exit interviews with clients and provide extra confidentiality assurance to clientele and their families when they give feedback on the care they received during their stay in the facility, since they may not be professionally trained health workers. The other team member(s) were experienced research data collectors.

#### Development of protocols and data collection instruments

Following agreements between investigators from the three countries and the UNICEF coordinating team on common implementation strategies, procedures for data collection, cadre of staff to be used for the data collection, harmonized training, and quality control mechanisms, the teams developed a generic protocol and data collection instruments for the assessment. Individual countries then adapted these for ethical clearance in their respective countries.

The team also agreed to collect a set of core variables with uniform set of questions across all the sites in order to allow for generation of common progress indicators, pooling of data to increase statistical power and comparison of progress on QI across sites and regions. Six generic semi-structured questionnaires were developed and core variables were assembled into tables and shared with all three sites (Appendix 1). All sites were mandated to collect these core variables but could optionally collect additional data as may be required to meet the specific needs of their respective countries. For instance, the Bangladeshi government has already started quality improvement efforts in certain health facilities across the country whilst Tanzania also recently conducted a nationwide facility EmOC assessment. The two countries could therefore ask specific questions as follow-up to those initiatives that preceded the EMEN-QI implementation. There was the caution to limit the number of additional optional questions to avoid respondent fatigue during the interview with consequent adverse effects on the quality of the responses. The contents of the questionnaires are summarized under the data collection sub-section below.

#### Sample size, sampling and considerations for selection of data collectors

Appropriate sample sizes required for the assessment were determined based on scientific and pragmatic considerations because this assessment mirrors what will be done in routine health system settings when evaluating a programme. The teams ensured that the sampling was representative of various care providers, key decision-makers within facilities and facility users and took account of the facility type, staff strength, projected workload/caseload and balance between intervention and comparison facilities. Table 4 provides the sample sizes used for EMEN baseline assessments and the justification for these. For instance, we decided that if any facility has specialist obstetricians, neonatologists, paediatricians, neonatal nurses or experienced matrons on the maternity or newborn units, they should be included in the interviews. We, however, determined an absolute maximum number of staff required per category. Where the numbers of a cadre of health care providers exceeds the maximum number required for that type of facility, interview respondents were selected by simple balloting using the roll of the total number of that cadre of staff as the sampling frame.

**Table 3:** Selected health facilities for the EMEN-QI assessment by country, level, and designated project arm (intervention r comparison)

Country	Facility level	Intervention facilities	Comparison facilities
Bangladesh	District Hospital	Kurigram District Hospital	Gaibandha District Hospital
			Lalmonirhat District Hospital
	Upazila Health Complexes	4 Upazila Health Complesxes in • Nageshwari, • Raomari, • Phulbari & • Ulipur	8 Upazila Helath complexes: • 4 from Gaibandha and • 4 from Lalmonirhat
Ghana	Municipal/District Hospital	Bawku Municipal Hospital	Kassena-Nankana Municipal Hospital
		Bolgatanga Regional Hospital	Bawku West District Hospital
		Bongo District Hospital	Builsa North District Hospital
	Health Centres	5 health centres in • Mognori • Sumbrungu • Bongo Soe • Paga • Sirigu	5 health centres in • Wiaga • Kologo • Binaba • Tongo • Pwalugu
Tanzania	District Hospital	Ludewa District Hospital	Makete District Hospital
		Ilembula District Hospital	Ikonda District Hospital
	Health Centres	4 Health Centres in • Mlangali • Manda • Wanging’ombe • Kidugala	4 Health Centres in • Njombe • Imiliwaha • Uwemba (Njombe) • Uwemba (Makete)

All women who deliver in target facilities and are discharged during the period of the assessment were eligible for the client exit interviews. As described earlier, if the caseload exceeds the total expected sample size, a systematic random sampling method was used to select clients. A ratio of the projected caseload during the period of contact with the facility and the sample size was used as the sampling interval (say n). A simple ballot was then cast, on the first day of the assessment, to choose the first respondent from the first n respondents and then the n^th^ client after that first one was sampled for the interviews. Similar systematic sampling technique were being used for sampling patient folders during the data abstraction in large facilities.

#### Training and supervision

The team developed a common training manual to be adapted and used by each country. The manual included the background and rationale and explained the principles for the conduct of the assessment, providing detailed explanation on the requirements from each question to avoid ambiguity. Data collectors were trained using this manual across the sites with a plan to deploy these data collectors in teams. All members of a data collection team were grouped together in some aspects of the training to facilitate team cohesion and to define roles to the team members. Team members therefore learned and worked synergistically to maximize the output from the contact with the facilities.

The training was in two phases over seven (7) days. All data collectors participated in the 1st phase with the objective to understand the purpose and principles of the assessment, general interviewing techniques, consenting and consent form administration, personal comportment within the facility, confidentiality and respect for patients’ rights to privacy and ethical practices around health research in the facility. It concluded with approaches to administering EMEN baseline assessment questionnaires with particular emphasis on those that do not require clinical interpretation or judgement e.g. client exit interviews, assessment of the existing facility structures, equipment, and the facility manager interviews. This first phase of training spanned three days; the initial two for the didactic and interactive training on the above-mentioned modules. The first half of the 3rd day was used to pilot the baseline assessment module in pre-selected pilot facilities. In the second half of the 3rd day, the assessment teams discussed the results of the pilot and fine-tuned the implementation strategy accordingly.

The second phase training lasted four days and focussed on the questionnaires that have technical content that required the clinical skills of health professionals’. The clinicians within the teams were taken through the clinical vignettes, observation of client-provider interactions and review of medical records of patients. Like the phase one, the interactive theoretical session was carried out over two and half days and the 2nd half of the third day was used for the pilot. After the training in the clinical modules during which the non-clinicians were excused to absent themselves from, the entire training participants were reconvened on the 4th and final day. They participated in the discussions around the experiences during the pilot by the clinicians and logistical planning for the completion of the phase two forms including strategies to retrieve of patient records, partographs and caesarean section records whilst simultaneously avoiding undue interference in the care for the client.

Specialised additional training was provided to team leaders for each facility or district to conduct quality control checks on the assessment process including facility entry, daily forms review, conflict resolution between data collectors and facility staff, data reporting and transmission processes to the central data entry centre and support for individual team members to promote unity.

#### Data collection

Six semi-structured questionnaires were developed to cover the criteria set out in the ten EMEN-QI standards. They directly address the four objectives derived from the assessment framework (Fig. 1) with each form tailored to collect data on one of the running headers (A to G) in the framework. Relevant and tested questions from existing tools such as Averting Maternal Death and Disability (AMDD) EmOC assessment tool, WHO’s Service Availability and Readiness Assessment (SARA), Ghana Newhints study facility assessment tools and unpublished documents on assessment of dignified and respectful care for mothers around childbirth. Input into the core variable tables were derived from these tools and they also helped in the formulation of the questions. Questionnaires for the EMEN assessment were annotated with instructions and guidance statements for data collectors on how best they should fill specific sections or questions on the forms. Where aspects of care content being assessed require observation rather than asking the respondent (such as the cleanliness of the toilet and the environment), the form provided italicised prompts for the data collectors to do so.

#### Content of the data collection forms

Table 5 summarizes the content of each data collection form. Essentially, form 1 assesses the availability of the necessary infrastructure to support the provision of quality care – the first step in the conceptual framework in Fig. 1. Senior facility managers and heads of various units assisted in the completion of form 1. Form 2 assesses facility policies and guidelines and data collectors obtained hard or soft copies for desk review, where available, to validate the information provided. Form 3 was administered to selected health professionals who directly care for pregnant women and/or their newborns. The form was administered on the maternity or newborn care ward of the facility or any suitable place selected by the respondent. Forms 4 and 5 required the data collectors to observe or obtain data on sets of maternal and newborn complications (from diagnosis through management) shown in Table 6.

**Table 4:** Sample size considerations for the assessment

Assessment mode	Facility type	Sample size
Observations:	Health centres	All deliveries in HCs will be observed up to a maximum of 5 deliveries. However, any complicated pregnancy such as pre-eclampsia, antepartum haemorrhage, preterm labour will be observed
	Hospitals	3 delivery observations per facility every other day: one per morning, afternoon and night shifts and therefore a total of 21 per hospital over the 2 weeks. Complications e.g. antepartum haemorrhage, pre-eclampsia, eclampsia, preterm labour, premature rupture of membranes, chorioamnionitis, etc. will be prioritized.
Health worker interviews	Health centres	Because these facilities are usually poorly staffed, we will aim to conduct interviews with 2–3 staff at the facility
	Hospitals	The following interviews will be conducted per facility: 1. Specialist Paediatrician/Obstetrician gynaecologist (1 each); 2. Medical Officers working in the labour ward (2–4); 3. Physician assistant (1–3); 4. Neonatal nurse (1); 5. Midwives (3–5); 6. Staff Nurse (2–3); 7. Other professionals who attend delivery in the labour ward (1–3)
Client exit interviews	All health facilities	All clientele who are admitted and discharged from the facility over the two weeks of the facility. All records of admissions and discharges over the period of the facility contact will be collected.
Records Review	Health centres	1. All deliveries in the past 3-months to the date of the visit will be reviewed. If less than 50 records are found, the review should be extended to cover the previous 6 months. 2. Partographs: Partographs for all delivery records picked will be reviewed but where rarely completed, the last 25 done with the previous 12 months should be reviewed. 3. Treatment records for all complications of delivery or newborn health will be reviewed.
	Hospitals	1. Deliveries in the 8 weeks to the date of the visit will be reviewed. This allows for capturing delivery up to mandatory postpartum visits to the facility. Where the previous 4 weeks coincide with a special event such as Ramadan etc., the other 4 weeks may compensate for the numbers and spectrum of cases. Assessors will review 25–30 delivery records each day starting with the most recent and working backwards. 2. Partographs: That for 20% of all deliveries in the previous 8 weeks will be reviewed. Selection will be by systematic sampling: each day, the reviewer will conduct a simple ballot to choose one out of the first five delivery records to be reviewed in the order in which the records were picked. Partographs of the 5th delivery after the one selected from the ballot will be reviewed. Where records are few, a minimum of 120 partographs should be reviewed. 3. Treatment records for all complications of delivery will be reviewed 4. All newborn health complications or illnesses will be reviewed 5. Records for all caesarean sections will also be reviewed.

The 6th form involved interviews with clientele and family or relatives who supported them during their stay in the hospital. It provides the most critical demand-side perspective of quality of care. There are internal validity challenges due to possible biases; for instance, women should be educated enough to be aware of what quality they deserve and hence judge the care they received based on that. On the other hand, since clients may perceive the interviews as coming from the health system, they may be tempted not to give a bad impression of the care they received for fear of the repercussions. The interviews were therefore conducted in a private setting after discharge from facility, by non-clinicians to provide assurance that the information they provide will not affect the care they received in the facility in the future and that all data they provide shall be handled in strict confidence. Again, clients judgement of the quality of care may depend on the birth outcomes and so mothers who had a stillbirth or early neonatal death may suggest bad quality of services in the facility and the contrary for those who had better outcomes. They were assured to be as encouraged as possible.

#### Data processing

The team leader of each facility manually checked all forms at the end of each day of work in the facility to assess completeness. Where blanks and inconsistencies were detected, the form was refilled on the next day or whenever the respondent was available. Forms were being signed as complete by the team leader and batched to be submitted or transferred electronically to the central data management centre timely, mostly daily. The team leadership also maintained a serialised log of all forms transferred.

All paper-based forms were independently double-entered, verified, and cleaned. This included range and consistency checks and inter-table consistency checks to ensure complementarity of data from all the different sources. Any queries identified were resolved promptly by the study coordinators, including re-visits to facilities or respondents, and the database updated accordingly. Cleaned data are being transferred into Stata statistical software (StataCorp., Texas, USA) for quantitative analysis. Back-up copies of the cleaned data were saved to dedicated study servers and on password-protected study external drives.

#### Statistical analysis

The analysis will assess designation of the facilities into BEmOC and CEmOC based on signal functions. The team also identified and proposed signal functions (Table 7) for basic and comprehensive emergency newborn care in facilities. The analysis will attempt to classify facilities by these criteria in order to determine whether there exists any correlation between emergency obstetric and newborn care functions within facilities. If such correlation exists and is strong, it may suggest that, granted our signal functions for the newborn are valid, the provision of EmOC simultaneously covers the provision of adequate emergency care for newborns. On the contrary, if such correlation does not exist or is poor, it may suggest that the provisions for EmOC has neglected care for newborns or vice versa.

**Table 5:** Data collection forms used in the EMEN baseline facility quality of care assessment

Form no	Respondent	Purpose & Content of the form
1	All departments for direct observation	The form was used to - assess the physical infrastructure in the facility for maternal and newborn care - obtain an inventory of all equipment to determine whether they were functioning well or not, the last time they were calibrated and - ascertain the expiry dates on some key drugs used for maternal and newborn care emergencies such as magnesium sulfate and antibiotics.
2	Facility manager	The form involved - desk review of facility protocols and policies for maternal and newborn care, - recruitment and placement procedures, - refresher training and workload management, - referral systems, - facility mechanisms for providing dignified care and abuse response as well as existence and activities of quality improvement teams.
3	Professionals providing care for mothers and newborns	It assesses - any formal and refresher training care providers received to validate the data provided by the superintendent in the form 2. - knowledge and care practices around care for mothers and babies in case of complications and - clinical skills directly through the administration of a clinical vignette to assess their knowledge and practices around selected maternal and newborn care emergencies. Their practices related to diagnosis and management of pre-eclampsia, obstetric haemorrhage, very low birthweight infant, perinatal asphyxia and hypothermia with be assessed in these vignettes.
4	Direct observation	The observation of client-provider interactions will involve - visual assessment of the dynamics of the interactions, - listening to the communications. - transcription of records taken on women to assess which aspects of the care were recorded but not verbalised such as the BP measurement if taken
5	Direct abstraction	Assesses the - completeness of record keeping and - content of care captured on patient records. - completeness of record keeping during labour monitoring with partographs together with actions taken based on the progress of the labour. - practices around caesarean section including records of the indications for and the entire management given intra- and post-operatively.
6	Mothers and companions	Assesses women’s perceptions on the content and quality of care including insights into whether - they thought the care was dignified, - they paid for any of the services and - any service was withheld because of non-affordability. It also assesses their overall impression about the facility and the quality of care they received.

**Table 6:** Selected maternal and newborn health conditions to be observed as part of the assessment of the EMEN facility quality of care

1. Management of complicated and uncomplicated labour,	
2. Management of complicated delivery (two of the following cases APH, preterm labour [with or without PROM], obstructed/prolonged labour, pre-eclampsia/eclampsia & PPH – this cannot be known at the time of labour)	
3. Immediate newborn care – routine and for a baby with foetal distress during labour,	
4. Immediate postnatal care within 24 h (before discharge from the facility) and	
5. Care of low birth weight (< 2000 g) or preterm baby	
6. Routine care for mother and baby who remain in hospital because of infection or caesarean delivery.	
7. Care of sick newborns

**Table 7:** Proposed Newborn Signal functions for Emergency Newborn Care

Type	Emergency Newborn Care Signal functions	
Basic	1. Essential Newborn Care - *Drying thoroughly* - *Skin to skin contact* - *Delayed cord cutting* - *Early initiation and exclusive breastfeeding*	- *Cord care* - *Eye care* - *Vitamin K* - *Weight* - *Temperature*
	2. Resuscitation - *Clearing of airway* - *Stimulation* - *Bag and mask ventilation*	
	3. Kangaroo mother care - *Dedicated space (optional): Records review for mothers who were kept in facility for more than usual with evidence of KMC (Prolonged STSC, EBF/feeding support, growth monitoring (weight))*	
	4. Management of suspected sepsis - *(Injectable) antibiotics (Ampicillin/Penicillin and Gentamicin)* i. *Availability* ii. *Administration*	
Comprehensive (Additional functions)	5. Newborn Intensive Care Unit or equivalent with - *Incubators (open) or radiant warmers* - *Phototherapy*	
	6. Advanced resuscitation - *Oxygen (blended)* - *Intubation* *Optional for preterm births* - *Nasal CPAP* - *Surfactant*	
	7. Advanced antibiotics (3rd generation Cephalosporin or higher) administration	
	8. IV fluid administration - *With infusion pump/perfuser*	
	9. Feeding (NG-tube)	
	10. ACS administration	

The baseline analysis will also assess how care provision in each individual facility as well as type (hospital, health facility or dispensary) meets each of the criteria in the EMEN standards and criteria. It will provide information on key performance indicators that will need to be tackled in the implementation of the QI model. Data will be reduced and represented with tabular, numerical and graphical measures. Means (and standard deviations) will be estimated for normally distributed continuous data and median (Interquartile range, IQR) for skewed data. Associations will be tested using chi-squared and differences in proportions with t- or z-tests.

Qualitative data will be transcribed into Microsoft Word and transferred into appropriate analytical software for analysis. Analysis will involve repeated reading of transcripts for identification of themes. Data will then be coded to these themes and the analysis will involve assessing relationship between these themes.

#### Coordination of the study across sites

UNICEF is coordinating the harmonization and monitoring the conduct of the baseline assessment across the sites. Having agreed on a uniform tool for the assessment, UNICEF collaborated with study teams from the respective countries to support the training and initiation of the data collection across the sites. All final versions of forms being used by teams in their respective countries have been shared with UNICEF. Principal investigators have and continue to provide progress reports and participate in regular (initially weekly and later fortnightly) telephone conferences with the UNICEF coordinating team and to harmonize decision making around the conduct of the assessment. An expert from UNICEF who supported the training and initiation also monitors the data collection, processing and will support in the analysis of the data. The UNICEF consultant conducted site initiation visits to all three sites at the inception of the assessment to assess their preparation, participate in the training of the data collectors and initiated the data collection in the study using a standardized implementation timelines and monitoring checklist.

#### Data monitoring and ethics committee (DMEC)

A five-member DMEC whose members will have expertise in epidemiology and medical statistics (including monitoring and Assessment of complex interventions), obstetrics, maternal and newborn health and quality of care within facilities. This committee met once prior to the onset of the baseline study to examine the assessment conduct and advised the study management teams on technical issues related to the study. One key recommendation from the committee was that the assessment should not be seen or referred to as a full evaluation and they advised on the content of the core variable tables, considering relatively short implementation time frame of 24 months.

#### Use of findings

Baseline assessment findings will identify the key components of quality and will stratify them into short, medium, and long term based on whether they can be implemented with limited immediate monetary costs and their potential to save maternal and newborn lives and prevent life-threatening complications that may result in disabilities. These findings will be promptly shared with EMEN-QI implementation teams to inform the direction of investment and resource allocation in the implementation of the quality improvement initiative. Briefing sessions will also be organised with heads of the health services and facility managers to devise strategies to expand QI initiatives to other facilities and to garner support for their implementation.

With coordination by UNICEF, forms/questionnaires, manuals, and methodologies used for the conduct of these baseline assessments as well as lessons learned will be made available to other countries for review and where findings are deemed applicable to any country’s system, they could be applied with minor modifications. Webinars, workshops and technical support will be provided to other countries through a formalised network to maximise the use of these resources.

## Discussion

With the publication of the WHO guidelines on *Standards for Improving Quality of Care for Mothers and Newborns in Health Facilities*, countries especially in low- and middle-income settings, will commence preparations to adapt these guidelines for implementation in health facilities. The evidence for these guidelines were not generated from the settings where the implementation is most critical to save lives of mothers and newborns. [12] There is oftentimes a gap between development of evidence-based interventions and their successful implementation. [19] Implementation needs to be informed with experiences from similar settings through structured knowledge translation including support for countries to adapt the recommendations into their specific contexts and build in metrics to assess impact.

The approach for this study has many strengths: 1) the theoretical framework covers all aspects of care quality improvement from inputs, through processes to possible outputs and outcomes. 2) assessment combines strategies that address both the content and the documentation of care delivered to clientele. This is a very important since evidence-based decision making is often hampered by availability of data in these LMICs. 3) it assesses both demand-side and supply-side perspectives on quality and therefore addresses dignified and respectful care provision as a key ingredient of quality from users and providers. 4) harmonization of the implementation across the sites in sub-Saharan Africa and South Asia presents a unique advantage since situations depicted in the facilities covered in these test countries’ baseline assessment may be similar to those in facilities in many LMICs. These are the settings where the gaps in current quality of care and what is optimal are largest with consequences on mortality and morbidity for mothers and babies. 5) manuals and tools used for the assessment pulled together best practices from those of WHO’s SARA and those used in robust research settings. This increases the external validity of the contents of the tools and, by extension, the some of the findings from the study.

Many such assessments collect reported practices through interviews and vignettes. In this framework, the study team directly observed practices and made a judgement of the quality of data capture, quality of interactions and overall quality of care and this is very important and unique. Although the findings are subject to bias in that health workers who know they are being observed may modify their practices towards what they think is most acceptable, historical review of records, for instance, will show whether the content of their data capture matches what they regularly do. Such differences will be important to highlight in implementing QI interventions since it demonstrates the potential and capability of health providers to provide better quality of care under whatever conditions they work.

The major limitations of the strategy include its cross-sectional design. The effect has been lessened by the review of records and vignettes to test knowledge and previous practices. The short duration of contact with facilities may also not allow for observation of key complications for maternal and newborn health especially in facilities where caseloads are small. This short duration is however pragmatic if the assessment is not to unduly delay implementation of QI interventions or interrupt care provision to clientele. Also, since the assessment is being carried out in specific health facilities in specific regions of the respective countries, the findings may not be fully representative of facilities within the countries let alone other countries with varying health systems capacities, inputs and processes. The content is comprehensive but the data may be overwhelming. It will therefore require careful analysis to stratify how the findings will be used. In these test countries, the findings are being looked at based on the ability to implement suggested recommendations in the short, medium or long terms considering how much capital needs to be invested to effect the required changes.

In conclusion, whilst the global momentum around quality of care is reaching its peak, global public health must understand that policy windows to support these interventions, which may be costly, within countries may be short-lived. [19] This strategy and framework provides a very useful model for supporting country implementation of the new WHO standards. It will serve as a template around which countries can build quality of care assessment strategies and metrics to inform their health systems on the effect of QI interventions on care processes and outcomes. Whilst a lot can be done to save the lives of mothers and babies in LMICs through concurrent strategies to facilitate universal coverage of care at optimal quality, health systems in LMICs should not unduly delay implementation and must act now before known threats [20] from competing challenges choke progress.

## Abbreviations

BEmOC or CEmOC: Comprehensive or Basic Emergency Obstetric Care
BMGF: Bill and Melinda Gates Foundation
DMEC: Data Monitoring and Ethics Committee
EMEN: Every Mother Every Newborn
ENAP: Every Newborn Action Plan
GHS: Ghana Health Service
ICDDR,B: International Centre for Diarrhoeal Diseases Research, Bangladesh
LMIC: Low- and middle-income country
MBFHI: Mother and Baby Friendly Health Facility Initiative
MNCH: Maternal, Newborn and Child Health
NHRC: Navrongo Health Research Centre
NIMR: National Institute of Medical Research
QI: Quality Improvement
QoC: Quality of Care
UHC: Universal Health Care
UNICEF: United Nations Children’s Fund
WHO: World Health Organization
